# Advancing Universal Health Coverage for refugees and migrants: highlights from the fifth edition of the Global School

**DOI:** 10.1016/j.lana.2024.100983

**Published:** 2024-12-27

**Authors:** Candelaria Araoz, Claudia Marotta, Santino Severoni

**Affiliations:** Health and Migration, Division of Universal Health Coverage and Healthier Populations, World Health Organization, Geneva 1202, Switzerland

Universal Health Coverage (UHC) ensures that all individuals and communities receive essential health services without financial hardship.^1^ The United Nations’ 2023 high-level meeting reaffirmed the global commitment to UHC, emphasizing national ownership and government responsibility in prioritizing it.^2^ Achieving UHC is firmly agreed to be an indispensable goal in country health system agendas and policies, and as public health approach for population’s better health outcomes. This vision is unattainable without addressing the unique needs of refugees and migrants.

The significance of this challenge has grown, with 281 million international migrants in 2020^3^ and 123 million forcibly displaced individuals recorded by 2024.^4^ Migration profoundly impacts health outcomes due to barriers like limited healthcare access, poor living and working conditions, and social discrimination. These factors exacerbate vulnerabilities, leading to poorer health outcomes for migrants compared to host populations.^5^ Addressing these challenges requires tackling the social determinants of health and integrating inclusive policies, aligning with UHC’s core principle of equity and the global right to health for all.

In this context, the 5th edition of the WHO Global School on Refugee and Migrant Health convened in Colombia from December 2–6,^6^ under the theme “*Advancing Universal Health Coverage for Refugees and Migrants: From Evidence to Action”.* Over 3000 participants engaged virtually to translate knowledge into sustainable policies and practices addressing migrant and displaced populations' health needs, fostering inter-country and inter-regional knowledge exchange through shared country experiences.

## Why Colombia?

Colombia’s approach to integrating migrants into its health system serves as a powerful model for inclusive policies that drive both social and economic benefits for migrants and host communities. With 1.5 million migrants receiving health insurance cards—500,000 of whom contribute financially—Colombia has strengthened its national health system while ensuring equitable healthcare access. Using a mixed financing model of contributory and subsidized schemes, the country balances inclusivity with financial sustainability, enabling employed migrants to contribute while protecting the vulnerable. This dual approach improves public health outcomes, fosters social cohesion, and enhances health system resilience, offering valuable lessons for global health and migration policy frameworks. This experience may offer a replicable blueprint for other countries facing similar challenges.

## WHO Fifth Global School on Refugee and Migrant Health: key takeaways

The five-day event, organised by WHO’s Health and Migration in collaboration with PAHO and Colombia's Ministry of Health and Social Protection, convened policymakers, researchers, healthcare providers, and civil society representatives. High-level and keynote speakers led discussions exploring the “mechanics” of advancing UHC for refugees and migrants. The following key messages emerged:

1. **Promoting Inclusive Primary Health Care for Refugee and Migrant Health Needs and Rights**
Primary health care (PHC) serves as the backbone of inclusive health systems, often serving as the first point of contact for refugees and migrants, ensuring access to essential services. PHC-oriented health systems promote equity by addressing diverse needs through preventive care, community-based interventions, and culturally sensitive approaches. Collaborative efforts between governments, agencies, and communities are essential for developing migrant-sensitive PHC initiatives towards advancing UHC.
2. **Managing the Continuum of Care for Non-Communicable Diseases (NCD) During the Migration Cycle**
Integrating NCD services, including mental health care, within PHC are essential for meeting the needs of refugees, migrants, and displaced populations. Overcoming access barriers requires legislative, financial, and operational reforms. Particular attention must be given to high-need conditions such as diabetes, renal failure, and cancer, which pose unique challenges for mobile populations.
3. **Including Migrants and Refugees in Comprehensive Health Financing Strategies**
Healthcare for migrants should be reframed as a global public good, requiring a shift towards supranational solutions such as global UHC funding pools. Cross-border financing mechanisms, including portable health benefits and extended health insurance, ensure continuity of care. Innovative approaches like supranational contributions and global taxation can ease the burden on low- and middle-income countries, promoting equitable resource distribution.
4. **Closing the Gap Between Research and Policymaking to Better Address the Health Needs of Refugees and Migrants**
Science and technology must be harnessed as public goods to promote equity in addressing the health needs of refugees and migrants. Incorporating their lived experiences ensures relevant solutions. Bridging the evidence-policy gap requires investing in health information systems for timely, disaggregated data while prioritizing research oh health and migration in high-burden regions through domestic investment and international partnerships.
5. **Universal Health Coverage Throughout and Beyond Migrant Health Emergency Crises**
Effective emergency responses require dual planning to meet immediate needs while integrating solutions into long-term health systems. Strengthening capacity, fostering partnerships, and ensuring equitable resource distribution are essential. Scalable and sustainable interventions, tailored to the cultural and linguistic needs of migrants, ensure broader system-wide solutions. Emergency response data must transition seamlessly into national systems to inform long-term public health planning for refugees and migrants.

## Conclusion

Achieving Universal Health Coverage requires inclusive policies that address the unique needs of refugees and migrants, with a focus on equity and sustainability. As migration continues to rise globally, sharing experiences and lessons—such as Colombia's innovative approach—is essential for shaping effective frameworks and policies. The WHO Global School on Refugee and Migrant Health serves as a pivotal platform for fostering collaboration, promoting knowledge exchange, and advancing health equity for these populations. Resources from this and previous editions remain available on OpenWHO, ensuring sustained momentum and actionable insights for driving progress.

## Contributors

CM contributed to writing the original draft as well as reviewing and editing the manuscript. SS and CA participated in reviewing and editing the manuscript. All authors reviewed and approved the final version of the manuscript.

## Declaration of interests

Authors declare no conflict of interest.

## References

[1] Boerma T., Eozenou P., Evans D., Evans T., Kieny M.P., Wagstaff A. (2014). Monitoring progress towards universal health coverage at country and global levels. PLoS Med.

[2] United Nations General Assembly (2023). https://digitallibrary.un.org/record/4025279.

[3] United Nations Department of Economic and Social Affairs, Population Division (UN DESA) (2021). https://www.un.org/en/desa/international-migration-2020-highlights.

[4] United Nations High Commissioner for Refugees (UNHCR) Refugee data finder 2024. https://www.unhcr.org/refugee-statistics.

[5] World Health Organization (WHO) (2022). https://www.who.int/publications/i/item/9789240054462.

[6] World Health Organization (WHO) Fifth global School on refugee migrant health (fifth WHO global School on refugee and migrant health 2-6 December 2024. https://www.who.int/news-room/events/detail/2024/12/02/default-calendar/fifth-who-global-school-on-refugee-and-migrant-health.

